# Display-Semantic Transformer for Scene Text Recognition

**DOI:** 10.3390/s23198159

**Published:** 2023-09-28

**Authors:** Xinqi Yang, Wushour Silamu, Miaomiao Xu, Yanbing Li

**Affiliations:** 1 College of Computer Science and Technology, Xinjiang University, No. 777 Huarui Street, Urumqi 830017, China; 2 Xinjiang Laboratory of Multi-Language Information Technology, Xinjiang University, No. 777 Huarui Street, Urumqi 830017, China; 3 Xinjiang Multilingual Information Technology Research Center, Xinjiang University, No. 777 Huarui Street, Urumqi 830017, China

**Keywords:** visual information, linguistic knowledge, transformer, scene text recognition, cross-modal attention

## Abstract

Linguistic knowledge helps a lot in scene text recognition by providing semantic information to refine the character sequence. The visual model only focuses on the visual texture of characters without actively learning linguistic information, which leads to poor model recognition rates in some noisy (distorted and blurry, etc.) images. In order to address the aforementioned issues, this study builds upon the most recent findings of the Vision Transformer, and our approach (called Display-Semantic Transformer, or DST for short) constructs a masked language model and a semantic visual interaction module. The model can mine deep semantic information from images to assist scene text recognition and improve the robustness of the model. The semantic visual interaction module can better realize the interaction between semantic information and visual features. In this way, the visual features can be enhanced by the semantic information so that the model can achieve a better recognition effect. The experimental results show that our model improves the average recognition accuracy on six benchmark test sets by nearly 2% compared to the baseline. Our model retains the benefits of having a small number of parameters and allows for fast inference speed. Additionally, it attains a more optimal balance between accuracy and speed.

## 1. Introduction

The first step in the scene recognition task involves detecting text regions in natural images, regardless of their shape. These identified regions are then cropped and further processed to extract their text content. One of the primary goals of Scene Text Recognition (STR) is to accurately recognize consecutive characters from the extracted regions [1,2]. STR finds its usefulness in various applications, including reading road signs, billboards, logos, and printed shirts. The practical significance of STR can be observed in autonomous driving, augmented reality, retail, education, and devices designed for the visually impaired individuals [3]. Unlike traditional Optical Character Recognition (OCR), which predominantly deals with uniform text attributes, STR encounters numerous challenges, including different font styles, varying orientation, diverse text shapes, uneven illumination, occlusion, and blurring. Furthermore, the images obtained from natural environments often exhibit noise, blurriness, distortion, or warping, making STR an immensely important yet highly demanding problem.

**Figure 1:**
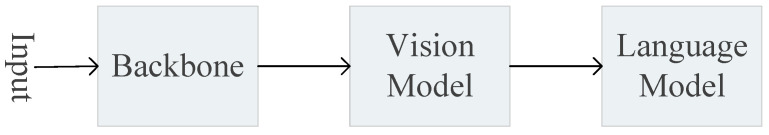
Two-step architecture of the visual language model.

STR primarily involves a visual task. However, when faced with unreadable portions in the image, e.g., occlusion or distortion, relying solely on image features is insufficient for accurate inferences. Scene text images consist of two levels of content: visual and linguistic information. However, the visual model lacks linguistic capabilities and merely focuses on the visual texture of characters, neglecting the linguistic information [4]. Incorporating linguistic knowledge into STR models has become a key research focus, as it enables the model to reason about characters based on context [5]. Numerous approaches have explored how to integrate linguistic knowledge into STR models, drawing inspiration from Natural Language Processing (NLP) techniques [6,7]. Recent studies have shifted their attention towards assisting scene text recognition by acquiring linguistic information [4,8,9,10]. Hence, the prevalent trend in recent methodologies is the adoption of a visual and linguistic modeling two-step framework, see [11] (as shown in Figure 1). In this approach, the visual model exclusively emphasizes the textual appearance of characters, neglecting any linguistic aspects. In contrast, language models use language learning structures such as Recurrent Neural Networks (RNNs) [12], Convolutional Neural Networks (CNNs) [13], and transformers [4] to infer the associations between characters.

Despite the favorable recognition outcomes garnered by these approaches in subsequent research, certain challenges persist. Language models impose an incremented computational burden, and numerous contemporary methods employ intricate bidirectional inference architectures to acquire more reliable linguistic insights. Regrettably, this exacerbates the computational load of the models, significantly curbing their efficacy in real-world scenarios [10,12,14]. In addition, an optimal STR model must prioritize not only recognition precision but also model velocity and computational efficacy.

In the work of this paper, we built a DST model based on DeiT-tiny and designed a semantic module and a semantic visual interaction module to allow the model to learn linguistic knowledge actively. Additionally, our model prioritizes computational efficiency, resulting in higher accuracy, faster speed, and reduced running costs.

## 2. Related Work

The study of STR has been the focus of extensive research over a considerable period [1,2,15]. Significant advancements have been accomplished in scene text recognition, owing to the advancements in deep learning [16,17,18]. STR techniques can be broadly categorized into semantic-free and semantic-aware methods based on their utilization of linguistic information.

### 2.1. Semantic-Free Methods

Earlier methods were based on semantic-free methods. These methods focused on extracting visual characteristics from an image to identify similarities, without explicitly considering the linguistic relationships between characters. Bai et al. proposed a novel strategy that utilizes sequences to enhance the conventional approach of character segmentation. This approach effectively avoids the challenges related to character segmentation while simultaneously capturing the relationships between local image regions and their semantic information [19,20]. Building upon this, Shi et al. proposed an approach based on Connectionist Temporal Classification (CTC), where the visual features extracted by the CNN are reshaped into a sequence of features, and then loss modeling is performed by RNN and CTC. Subsequently, this sequence is subject to loss modeling by leveraging RNN and CTC [21]. In a subsequent study, He et al. introduced several techniques to enhance recognition accuracy. These strategies involve the design of deep convolutional recurrent networks [22], the integration of multiple RNNs [23], and the implementation of graph convolutional networks to guide CTC decoding instead of relying solely on RNNs [24], among others. Segmentation-based methods perceive recognition as a segmentation problem, either standard or modified, where every single character represents a specific category [7,25,26]. Nevertheless, these methods commonly necessitate character-level annotations, which can be challenging to acquire. Additionally, they exhibit sensitivity toward segmentation noise. In general, the recognition performance of semantic-free methods is limited as they often need to pay more attention to the contextual information of the text. In real-world application scenarios, understanding scene texts accurately usually requires incorporating both semantic comprehension and contextual information. To overcome these limitations of existing methods, it is necessary to utilize more in-depth semantic understanding and contextual information to enhance the accuracy and robustness of scene text recognition.

### 2.2. Semantic-Aware Methods

Semantic-aware methods, on the other hand, use linguistic rules to aid the recognition process [10,27,28,29]. In the study by Li et al., RNNs were employed to acquire knowledge of the sequential patterns within a sequence without requiring manual specification of N-grams [18]. On the other hand, Aster first employs a correction module prior to recognition, then utilizes RNNs to capture the linguistic properties of the previously predicted character [12]. However, the sequential and time-dependent nature of RNNs restricts both the computational efficiency and the performance of semantic reasoning [4]. To overcome this limitation, SRN introduces a global semantic inference module for purely linguistic modeling using a transformer unit [30]. This module takes the predictions of the visual model as input and predicts the relationships between characters to refine the recognition outcomes [4]. Fang et al. introduced a novel architecture that integrates visual and linguistic modeling by utilizing CNNs [13]. The R2AM technique applies recursive CNNs to extract relevant features and incorporates LSTMs as implicit language models to enhance linguistic modeling [18]. JVSR proposes a multi-level decoder that refers to visual features multiple times to enhance semantic features [31]. Specifically, it is based on a multi-level RNN-attention decoder where each level generates an output sequence and utilizes visual features to update each hidden state. MATRN introduces cross-modal transformer modules to explore the interaction between visual and language features, incorporating semantic features and visual features for enhanced interaction, providing a more comprehensive and accurate text recognition method for better recognition performance [32]. LevOCR further investigates how to effectively fuse visual and language features [33]. In order to learn internal language models at the visual level, VisionLAN introduces a visual inference module that randomly masks visual features corresponding to characters during the training process [11]. MGPSTR proposes a novel approach to improve the performance of scene text recognition by learning implicit context to enhance the model’s robustness and accuracy [34]. LPV improves the understanding and recognition capability of scene text by introducing a technique called Linguistic More, which utilizes additional linguisitic features and enhances the processing of language information, enhancing the efficiency and accuracy of scene text recognition [35]. ABINet proposes an autonomous, bi-directional, and iterative language modeling approach, improving recognition accuracy by iteratively optimizing the language model [36]. PARseq proposes a scene text recognition method based on the replacement autoregressive sequence model, the core idea of which is to model text sequences using autoregression, which brings a new idea and methodology for the research and application in the field of scene text recognition [3]. Although these methods have achieved commendable results in scene text recognition tasks, introducing additional language models has resulted in excessive model parameters and increased inference time and complexity. Therefore, finding a better balance between the computational cost of language models and the accuracy of model recognition is also a challenging problem.

Our work relates to semantic-aware methods, designing a textual model and semantic visual interaction module. However, what distinguishes our approach from most semantic-aware methods is our focus on achieving higher accuracy with fewer parameters. We prioritize the balance between model accuracy, speed, and parameter quantity. Unlike semantic-free methods, we actively introduce semantic information to assist scene text recognition, leveraging contextual cues to enhance character recognition accuracy. The experimental results show that our model excels in inference time, number of model parameters, and algorithm complexity, which are only 15.9 ms, 8.57 m, and 5.919 G, respectively, and has a relatively better recognition effect.

## 3. DST Model

**Figure 2:**
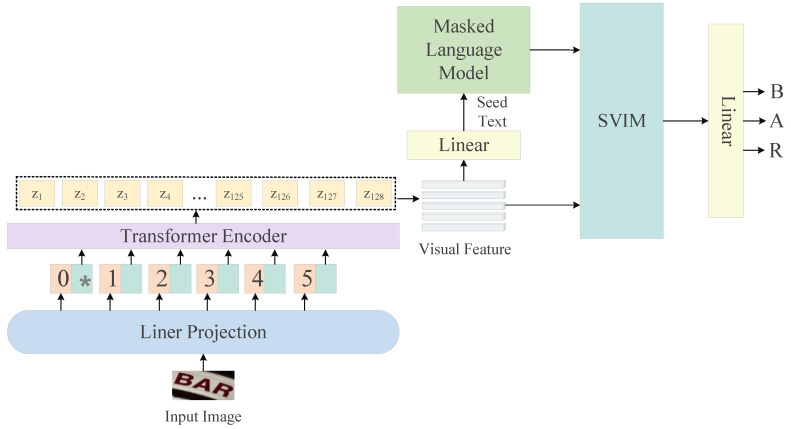
The model architecture of the proposed DST ("*" represents learnable class embedding).

In order for the model to obtain better recognition with less computational cost, based on the Vision Transformer model, we designed a DST model as shown in Figure 2. To control the parameter size of the model, the visual model uses a Vision Transformer to extract visual information. In the semantic branch, we designed a masked language model to help the model actively learn the language knowledge, which can reason about the characters based on the context and improve the recognition effect and robustness of the model. In addition, we build a semantic-visual interaction module to achieve interactive learning between semantics and vision. In this way, visual features can be enhanced by semantic information, which helps the model to find the characters to be recognized, thus improving the recognition effect of the model.

### 3.1. Image Processing

The DST model first passes the input image through a linear mapping, which consists of a convolutional layer with a convolutional kernel and stride that are both P×P (P×P is the patch size of the Vit (Vision Transformer)) and the resulting initial features are summed with a learnable positional encoding of the exact dimensions. The resulting vector sum is used as the input to the encoder.

### 3.2. Visual Model

**Figure 3:**
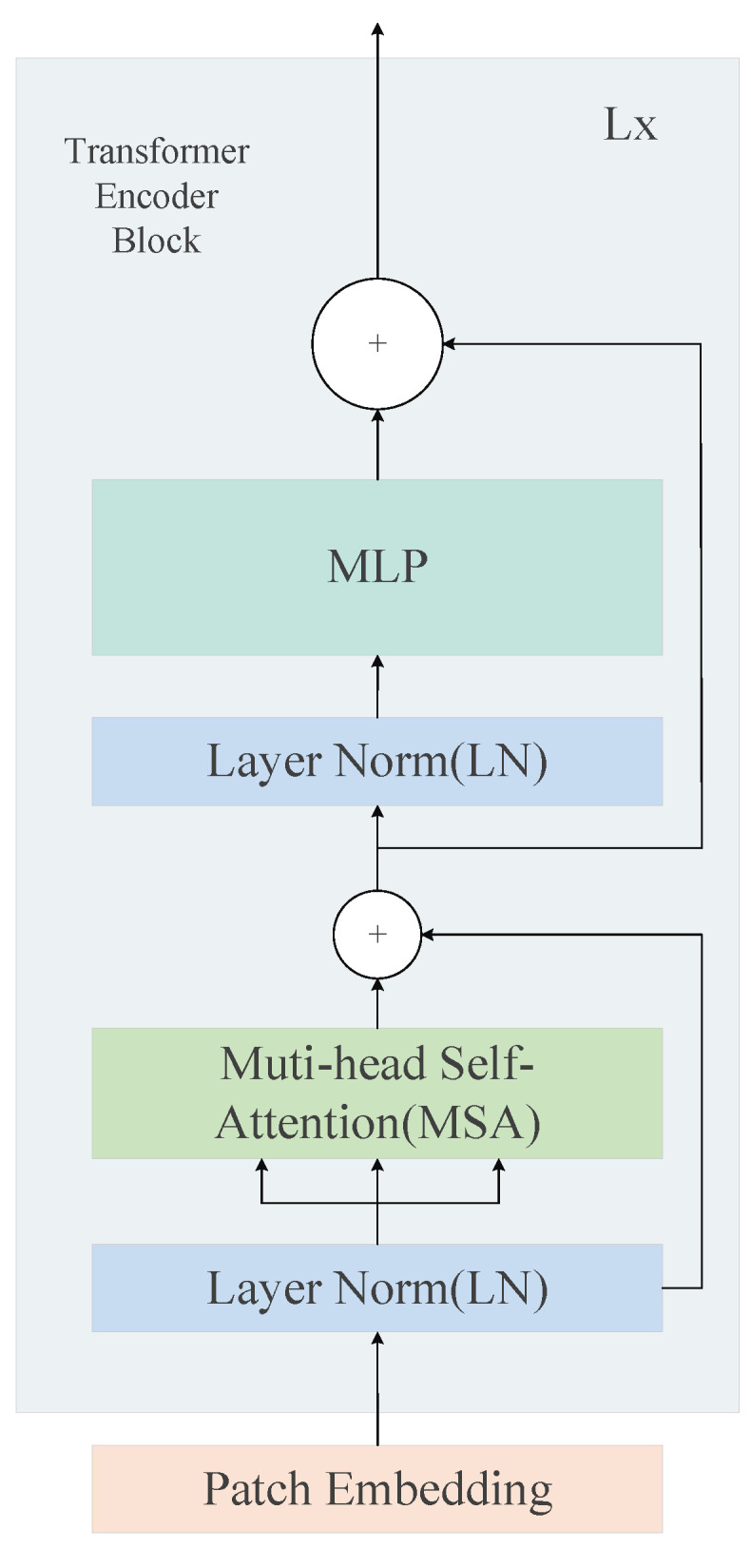
Transformer encoder for the L layer.

As shown in Figure 3, the vision model consists of a 12-layer Vision Transformer (Vit), which is a direct extension of the transformer to the image. Each Vit layer consists of a multi-headed self-attentive module, i.e., q = k = v. the image size of the input to our model is set to 32×128, and each input image $x\in R^{H\times W\times C}$ is divided into a series of 2D patches $x_{p}\in R^{N\times \left(P^{2}C\right)}$. The image size is H×W and the number of channels is C, while the dimensions of these block patches are P × P. The length of the generated patch sequence is 192. Each embedding adds a learnable positional embedding of the same dimensionality as it, and the sum of the two of them is taken as the input to the Vit encoder before being processed by the first Vit layer. The input to this encoder is:

(1) $$Z_{0}=\left[x_{class};x_{p}^{1}E;x_{p}^{2}E;\ldots ;x_{p}^{N}E\right]+E_{pos}$$

where $E\in R^{P^{2}C\times D}$ and $E_{pos}\in R^{(N+1)\times D}$.

Firstly, the input of each layer Vit passes through a layer normalization (LN) and then it continues through the Multi-headed Self-Attention layer (MSA) followed by one more layer normalization and Multilayer Perceptron (MLP). In addition, MSA establishes the interdependence between the feature vectors. The MLP consists of two linear layers of GELU activation functions to complete the feature extraction and the output obtained is used for residual concatenation.

The formula for the MSA module is:

(2) $$z_{l}^{\prime }=MSA\left(LN\left(z_{l-1}\right)\right)+Z_{l-1}$$

where L represents the encoder depth of the Vit.

The output of the MLP block is:

(3) $$Z_{l}=MLP\left(LN\left(z_{l}^{\prime }\right)\right)+z_{l}^{\prime }$$

The final output feature $Z_{l}\in R^{(N+1)\times D}$ is used as input to the semantic visual interaction module.

### 3.3. Masked Language Model

**Figure 4:**
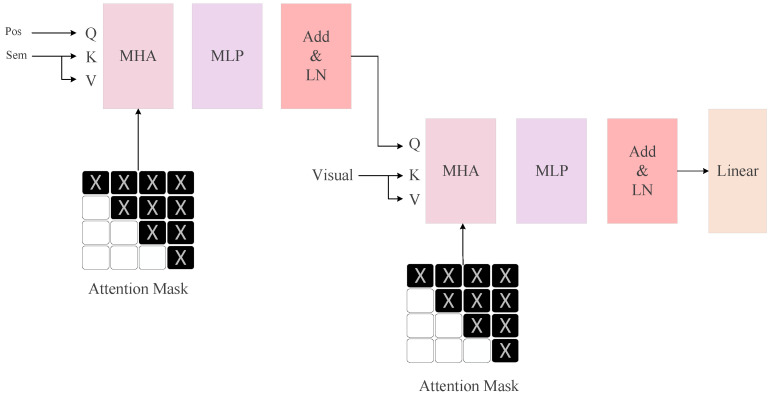
Details of masked language models and SVIM.

The model is more robust due to its ability to use semantic information to help understand visual cues in scene text recognition. Our model designs a masked language model with transformer units to extract semantic information. As shown in Figure 4, the multi-head attention module of this masked language model uses positional information as q. We first generate a sequence of vectors with a fixed constant for each position index dimension; otherwise it is 0. Then, a sinusoidal positional embedding is used with the same, followed by two MLP layers to obtain the positional embeddings, and the positional embeddings are computed using the following formulae.

(4) $$PE\left(pos,2i\right)=\sin\left(pos/{10,000}^{2i/d_{model}}\right)$$

(5) $$PE\left(pos,2i+1\right)=\cos\left(pos/{10,000}^{2i/d_{model}}\right)$$

Where pos represents the positional information, i is the character position, and d represents the dimension of the positional information. The value 10,000 in position encoding represents the length of the sine and cosine functions’ cycles, which provide unique encoding for different input positions. Choosing 10,000 is performed to maintain a certain level of periodicity and accommodate the dimensions of the hidden layers. In this PE matrix, the sine variable is inserted at even positions, while the cosine variable is inserted at odd positions. Then, the visual features extracted by Vit are passed through a linear layer to obtain the seed text, which is used as k and v in the multi-head attention module after word embedding. Meanwhile, we use a mask to prevent information leakage over time. The output obtained after the multi-head attention module is through the MLP layer and residual connection to obtain the extracted semantic information.

The output of the multi-attention module is:

(6) $$\mathrm{MultiHead}\left(Q,K,V\right)=Concat\left({\mathrm{head}}_{1},\ldots ,{\mathrm{head}}_{n}\right)W^{0}$$

(7) $${\mathrm{head}}_{i}=\mathrm{Attention}\left(Qw_{i}^{Q},Kw_{i}^{K},Vw_{i}^{V}\right)$$

where Q, K, and V represent the query, key, and value, respectively, and $W_{i}^{Q}$, $W_{i}^{K}$ and $W_{i}^{V}$ are the weight matrices of the ith attention head. The splicing function Concat combines the outputs of the different attention heads; in addition, the process uses a matrix of trainable parameters $W^{0}$.

The final output of the multi-head attention module is obtained by the residual connection of the results of the attention module with the input query. The resulting sum is then subjected to a normalization layer.

The output of the residual connection and the output of the normalization layer are:

(8) x=Q+Dropout(MultiHead(Q,K,V))

(9) $$S^{\prime }=\mathrm{LayerNorm}\left(\frac{\gamma }{\sigma }\left(x-\mu \right)+\beta \right)$$

During the process of training, the average and variance of the dimension vectors in the sample are represented by μ and σ, respectively, while γ and β denote the scaling factor and translation factor, respectively, obtained through learning.

The FeedForward Network (FFN) module performs feature extraction and the output of the FFN module is:

(10) $$\mathrm{FFN}\left(S^{\prime }\right)=W_{2}\mathrm{ReLU}\left(W_{1}\left(S^{\prime }\right)+b_{1}\right)+b_{2}$$

The weight matrix and bias parameters of the FFN, denoted as $W_{1}$, $W_{2}$, $b_{1}$, and $b_{2}$, are learnable parameter matrices. The final semantic information S is obtained by passing the results of the FFN layer through the Dropout layer, followed by residual concatenation, and finally through the normalization layer.

The output of the layer normalisation layer is:

(11) $$S=\mathrm{LayerNorm}(S^{\prime }+\mathrm{Dropout}\left(\mathrm{FFN}\left(S^{\prime }\right)\right))$$

### 3.4. Semantic-Visual Interaction Module (SVIM)

In order to establish the correspondence between visual semantics, we use semantic features as the query to interact with visual features, which is beneficial to help the model find the character to be recognized. For this purpose, we designed a semantic visual interaction module, as shown in Figure 4. The input to the semantic visual interaction module comes from the information extracted from the visual model and the masked language model. The multi-head attention layer will first extract this input, and the obtained extracted information will then go through the multi-layer perceptron, residual connection, and layer normalization to obtain the improved visual information. In the multi-head attention module, the query is the linguistic information extracted by the semantic module, and key and value are the visual information extracted by the visual model. Meanwhile, in the attention module, we use a mask to prevent the leakage of semantic information across time steps. The interaction between semantic and visual information is based on the semantic enhancement of visual information. Enhanced visual information is captured in Equations (6)–(11).

### 3.5. Character Prediction and Loss Calculation

Enhanced visual features are projected through linear projections to obtain character predictions.

(12) $$y_{i}=Linear\left(V_{L}^{i}\right)$$

For i = 1 *…* S. S is the maximum text length the model can predict plus two of the [GO] and [s] tokens.

The cross-entropy loss function is effective in driving model optimization for classification tasks. When the model-predicted results do not match the true labels, the cross-entropy loss function generates a more considerable loss value, which directs the model’s attention towards incorrect predictions. The backpropagation algorithm updates the model parameters to reduce the loss value. In multi-class classification problems, the cross-entropy loss function quantifies the quality of the model’s predictions for each class. The accuracy of the model across different classes can be evaluated by calculating the cross-entropy between the probability distribution outputted by the model and the true labels. When used with the softmax activation function, it effectively drives the model to predict the correct class with higher probability and continuously improves the classification performance during training. So, we use the cross-entropy loss function to calculate the loss.

(13) $$L_{CE}\left(y,t\right)=-\sum_{i=1}^{C}t_{i}\log y_{i}$$

The loss $L_{}$ is denoted by denoting the loss, the model output is denoted by y, the true labels are denoted by t, and the number of label categories is denoted by C. $t_{i}$ denotes the true label value of the ith category and $y_{i}$ denotes the predicted probability value of the ith category.

### 3.6. Methodology

#### 3.6.1. Datasets

As with most scene text recognition methods, the proposed network model is trained by using two publicly available synthetic datasets, namely MJSynth [37] and SynthText [38]. MJSynth has nine million synthetic text images, which use a combination of fonts and colors to render the text in a naturalistic manner into real images, thus generating a dataset of text images. The images presented in each instance showcase synthetic text in various forms, encompassing distinct textual content and text styles. Primarily designated for model training, this dataset, SynthText, comprises an extensive collection of seven million images. Notably, it encompasses examples featuring special characters. Each text image includes an authentic vertical text layout. While most characters within the images are in English, there are also genuine vertical columns of text in other languages. Unlike MJSynth, the text in SynthText is integrated into real-life scenes, including billboards, street signs, and road signs. We conducted extensive experiments on six standard benchmark datasets, including three regular text datasets, IIIT5Kwords, Street View Text, ICDAR2013, and three irregular text datasets, ICDAR2015, Street View Text Perspective, and CUTE80.

IIIT5K-words (IIIT5K) [39] is an extensive collection of 5000 images from natural scene images assembled through Google Image Search. This dataset comprises 2000 images designated for training purposes and 3000 for testing. The textual representations within these patches follow a consistent horizontal arrangement.

The StreetViewText (SVT) [40] dataset consists of 647 images extracted from Google Street View and precisely cropped for testing purposes. The majority of these images are in a horizontal orientation. However, it is essential to note that they are significantly affected by various issues such as noise, blurriness, and a low level of resolution.

ICDAR2013 [41] was a dataset consisting of 288 scene-true images. Additionally, it included 1095 images that were cropped from mall images. However, for testing purposes, only 857 images were used. The remaining images were discarded due to their inclusion of non-alphanumeric characters or having less than three characters.

The dataset ICDAR2015 [42] consists of word chunks extracted from incidental scene images taken from various angles. As a result, most word chunks within this dataset exhibit irregularities, such as being oriented, perspective, or curved. It comprises 4468 training data samples and 2077 test data samples.

StreetViewTextPerspective (SVTP) [43] is a dataset containing 639 images from side view snapshots in Google Street View. These images have significant perspective distortions and are specifically designed for model testing.

CUTE80 [44] includes 288 high-resolution images specifically designed for testing purposes. Among these images, a substantial portion consists of curved and irregular text.

#### 3.6.2. Experimental Setup

In our experiments, the dimensions of the input images were set to a height of 32 and a width of 128. For visual feature extraction, we used DeiT-tiny [45] as a module for the model, retaining the same experimental configuration as before, except for a different input image size. To enhance the data [46], we applied standard techniques for scene text images, such as blurring, noise, perspective distortion, and rotation. Our model training was performed using two RTX A5000 GPUs with a batch size 360 and one million iterations. The Adadelta optimizer [47] was chosen for optimization with an initial learning rate of 1. To fine-tune the learning rate during training, we used the cosine annealing LR [48] strategy.

## 4. Experiment

### 4.1. Proof of Concept for the DST Model

Since the DeiT-tiny pure vision model cannot utilize contextual information for assisted recognition in some low-quality images, we aim to improve the recognition effect of the model by building a masked language model to help the DST model use contextual information for scene text recognition. Moreover, to further utilize the semantic information extracted by the masked language model, we build a semantic visual interaction module to help the model recognize the character by using the interaction between semantic visuals. We conduct experimental analysis and ablation validation of the proposed framework in the following sections. Based on the results of the analyses, it is proven that our proposed framework is feasible and effective.

### 4.2. Comparison of Accuracy with Existing Methods on Six Standard Benchmarks

Our model is built on DeiT-tiny, an augmented model incorporating knowledge distillation and Vit. In our designed model, we use DeiT-tiny as a visual model to extract the visual information and enhance it with a semantic branch, semantic module, and semantic visual interaction module, which effectively enhances the robustness of the model and improves the recognition rate of the model. Table 1 compares our models with the most advanced scene text recognition methods. We have carefully analyzed and summarized the results obtained for each model on the six datasets. It can be seen that by comparing our model with other models, our model works best on three datasets, ICDAR2013, SVTP, and ICDAR2015, in addition to performing well in terms of accuracy on the other three datasets. Overall, our model improves by nearly 2% on average over the baseline. The DST model effectively improves recognition accuracy by adding the masked language model and the visual semantic interaction module.

### 4.3. Ablation Study

Our subsequent experiments are also based on the DeiT-tiny model. The findings presented in Table 2 illustrate that incorporating a module for semantic information into the baseline aids in enhancing the average accuracy by nearly 1%. This implies that the amalgamation of visual and semantic information is a practical approach to recognition. To make the recognition even more effective, we add a semantic-visual interaction module to the model, which enhances the recognition accuracy of the model through the interaction between semantic and visual information. Experiments show that our improvements are practical, and our model’s recognition rate improves by nearly 2% compared to the baseline.

### 4.4. Comparison of Inference Times and Model Parameters

By comparing with some classical language models, SRN is an approach that uses semantic reasoning to improve the accuracy of scene text recognition, and by introducing semantic awareness and reasoning modules, the system can better understand the contextual information of the text, thus achieving superior performance. Visionlan proposes a novel scene text recognizer that integrates text detection and recognition, and by using a visual language modeling network, the system can better exploit the ABINet is ability to accurately recognize text in various complex scenarios through comprehensive modeling of text contextual information and iterative optimization. MGPSTR uses a multi-granularity prediction approach, which fully uses local and global information about the text to achieve more accurate and robust scene text recognition. PARSeq uses an aligned autoregressive sequential model for PARSeq uses an aligned autoregressive sequence model for scene text recognition, which enables the system to better understand and recognize complex scene texts by dealing with changes in text order. As shown in Table 3, our model outperforms SRN and visionLAN in accuracy by 0.28% and 0.45%, thanks to our designed language model and semantic visual interaction module, and the size of the computational cost and the inference speed of our model are much better than those of SRN and visionLAN. Though compared with ABINet, MGP-STR, and PARSeq, our model is slightly lower in recognition accuracy, the computational cost of these models is, on average, about 4.3 times that of our model, and the inference speed of ABINet is much greater than that of our model. In terms of model complexity, only PARseq performs better; all other models have greater complexity than ours. Based on these data, it is evident that our model exhibits excellent performance both in terms of accuracy and computational cost.

## 5. Conclusions

In order to enable the scene text recognition model to actively learn linguistic knowledge and reduce the computational cost of the model, we designed the DST model based on DeiT-tiny. Firstly, we added a language branch based on the transformer and designed a masked language model to enable the model to learn linguistic knowledge actively, thus solving the problem that the model only focuses on visual texture without learning linguistic knowledge and the constructed semantic visual interaction module enhances the visual information through the interactions between linguistic vision. Secondly, compared with most of the language models with sizeable computational costs, our model has a minor computational cost of only 8.57 M parameters and achieved better recognition results and significantly improved the model’s performance. Compared to existing baseline models for scene text recognition, our model consistently performs well in terms of inference speed, recognition rate, and number of model parameters, making our model more practical and feasible for real-world applications in various scenarios.

## Figures and Tables

**Table 1:** Comparison with state-of-the-art methods.

Methods	Year	Datasets	IC13	SVT	III5K	IC15	SVTP	CUTE80	Avg
TRBA	2019	MJ+ST	93.6	87.5	87.9	77.6	79.2	74	84.6
TextScanner	2019	MJ+ST	92.9	90.1	93.9	79.4	84.3	83.3	88.5
DAN	2020	MJ+ST	93.9	89.2	94.3	74.5	80	84.4	87.2
SRN	2020	MJ+ST	95.5	91.5	94.8	82.7	85.1	87.8	90.4
RobustScanner	2020	MJ+ST	94.8	88.1	95.3	77.1	79.5	90.3	88.4
SAM	2021	MJ+ST	95.3	90.6	93.9	77.3	82.2	87.8	88.3
ViTSTR	2021	MJ+ST	93.2	87.7	88.4	78.5	81.8	81.3	85.6
VisionLAN	2021	MJ+ST	95.7	91.7	95.8	83.7	86	88.5	90.23
PIMNet	2021	MJ+ST	95.2	91.2	95.2	83.5	84.3	84.4	90.5
MGP-STR	2022	MJ+ST	95.7	93	95.6	83.6	89	88.5	91.3
PARSeq	2022	MJ+ST	95.7	92.4	96	83.1	88.7	90.6	91.4
baseline	-	MJ+ST	95.799	91.499	91.333	82.827	85.116	81.597	88.811
DST	-	MJ+ST	97.316	93.045	92.8	84.539	87.752	83.681	90.68

**Table 2:** Comparison of ablation experiments.

	IC13	SVT	IIIT5K	IC15	SVTP	CUTE80	Avg
baseline	95.799	91.499	91.333	82.827	85.116	81.597	88.811
+MLM	96.266	92.382	92.26	83.821	86.822	81.944	89.804
+MLM+SVIM	97.316	93.045	92.8	84.539	87.752	83.681	90.68

**Table 3:** Comparison of ablation experiments.

Methods	Year	IC13	SVT	IIIT5k	IC15	SVTP	CUTE80	Avg	Parm (m)	Speed (ms)	FLOPs (G)
SRN	2020	95.5	91.5	94.8	82.7	85.1	87.8	90.4	54.7	25.4	11.36
VisionLAN	2021	95.7	91.7	95.8	83.7	86	88.5	90.23	32.8	28	-
ABINet	2021	95.2	93.4	97	83.4	89.6	89.2	91.9	36.7	51.3	10.94
MGPSTR	2022	95.7	93	95.6	83.6	89	88.5	91.3	52.6	9.37	25.4
PARSeq	2022	95.7	92.4	96	83.1	88.7	90.6	91.4	23.8	11.7	3.25
ours	-	97.316	93.045	92.8	84.539	87.752	83.681	90.68	8.57	15.9	5.919

## Data Availability

The data that support the findings of this study are openly available in the public domain.
